# Cellular and network mechanisms of cholinergically-induced transition between gamma and sharp wave – ripple network states in the hippocampus

**DOI:** 10.1186/1471-2202-12-S1-P134

**Published:** 2011-07-18

**Authors:** Szabolcs Káli, Rita Karlócai, Norbert Hájos, Tamás F Freund, Attila Gulyás

**Affiliations:** 1 HAS - PPCU - SU Neurobionics Research Group, Budapest 1083, Hungary; 2 Institute of Experimental Medicine, Hungarian Academy of Sciences, Budapest 1083, Hungary

## 

In different behavioral states cortical networks show distinct dynamics, which may implement distinct computations. Switching between network states may be brought about by subcortical modulatory inputs, which affect both single cell and synaptic properties, but the actual mechanisms are currently unknown. Hippocampal activity in awake rodents is characterized by the behavior-associated alteration of theta-modulated gamma activity, associated with exploration, and sharp wave – ripple (SPW-R) activity, associated with alert stillness. We take advantage of the recent development of an in vitro slice preparation where these activity patterns either emerge spontaneously or can be induced by the application of neuromodulatory substances, to investigate, via the combination of electrophysiological and modeling tools, the link between changes in neuronal characteristics and the resulting switches in emergent network behavior.

By improving the preparation and maintenance conditions of in vitro slices, we managed to record activity patterns identical to in vivo described SPW-Rs and gamma activity from the CA3 area. Under baseline conditions the slices spontaneously generated SPW-Rs with characteristics similar to those observed in vivo. Mimicking the increased cholinergic tone during the theta-gamma state in vivo by the application of the cholinergic agonist carbachol (3-5uM, CCH), the SPW-R activity can be quickly (1-2 minutes) and reversibly converted into gamma activity. In the SPW-R state, activity levels were generally low, but activity in the network could transiently build up to an intensely activated, highly synchronous state. In the gamma state, activity levels were sustained at a high level, and synchrony increased rhythmically in each cycle, but never reached levels seen during SPW-Rs. In order to explain these qualitative changes in network behavior, we hypothesize that two network parameters change concurrently during the SPW-R to gamma transition, caused by the increasing cholinergic tone: 1) a cellular parameter: the excitability of principal neurons increases (this is responsible for the increased frequency of network activation), while 2) a network parameter: the strength of synaptic connections decreases (this limits the spread of activity in the network before inhibition terminates it).

We measured the effects of CCH on the excitability of single pyramidal cells and perisomatic interneurons, and determined how CCH changes the efficacy of transmission between these cell types in CA3. We confirmed that CCH increased cellular excitability (mainly in pyramidal cells), and found that CCH substantially decreased the efficacy of synaptic transmission in all connections examined, but it especially strongly attenuated excitatory synaptic transmission.

Finally, we developed a large-scale model of the CA3 area of a hippocampal slice, consisting of integrate-and-fire models of pyramidal cells and interneurons. Single cell and synaptic properties were determined experimentally in both network states. Our model confirmed that the measured cellular and synaptic changes are sufficient to explain the observed switching between the two types of network dynamics, and also suggested possible mechanisms for the initiation and termination of sharp wave events. it suggested that the initiation of sharp waves relies on random fluctuations of population activity, while the termination of sharp waves probably requires some novel form of slow negative feedback.

